# Imaging patterns of glioblastoma progression: Comparative analysis of sequential proton boost versus conventional photon therapy

**DOI:** 10.1093/noajnl/vdag116

**Published:** 2026-05-04

**Authors:** Ludwig Singer, Hanna Gött, Viet Duc Vu, Rita Engenhart-Cabillic, Fabian Eberle, Hildegard Dohmen, Alexandra Jensen, Stefan Lang, Marco Stein, Eberhard Uhl, Arnd Dörfler, Tobias Struffert, Manuel Alexander Schmidt

**Affiliations:** Department of Neuroradiology, University Hospital Erlangen, Friedrich-Alexander-University Erlangen-Nürnberg, Erlangen, Germany; Department of Neuroradiology, University Hospital Giessen, Justus Liebig University, Giessen, Germany; Department of Neurosurgery, Eberhard Karls University, Tübingen, Germany; Department of Neurosurgery, University Hospital Giessen, Justus Liebig University, Giessen, Germany, (H.G., M.S., E.U.); Department of Diagnostic and Interventional Radiology, University Hospital Giessen, Justus Liebig University, Giessen, Germany; Department of Radiation Oncology, University Hospital Marburg, Philipps-Universität Marburg, Germany; Marburg Ion-Beam Therapy Center (MIT), Germany; Department of Radiation Oncology, University Hospital Marburg, Philipps-Universität Marburg, Germany; Marburg Ion-Beam Therapy Center (MIT), Germany; Institute of Neuropathology, University Hospital Giessen, Justus Liebig University, Giessen, Germany; Department of Radiation Oncology, University Hospital Giessen, Justus Liebig University, Giessen, Germany; Department of Neuroradiology, University Hospital Erlangen, Friedrich-Alexander-University Erlangen-Nürnberg, Erlangen, Germany; Department of Neurosurgery, University Hospital Giessen, Justus Liebig University, Giessen, Germany, (H.G., M.S., E.U.); Department of Neurosurgery, University Hospital Giessen, Justus Liebig University, Giessen, Germany, (H.G., M.S., E.U.); Department of Neuroradiology, University Hospital Erlangen, Friedrich-Alexander-University Erlangen-Nürnberg, Erlangen, Germany; Department of Neuroradiology, University Hospital Giessen, Justus Liebig University, Giessen, Germany; Department of Neuroradiology, University Hospital Erlangen, Friedrich-Alexander-University Erlangen-Nürnberg, Erlangen, Germany

**Keywords:** AI, automated tumor segmentation, glioblastoma, perfusion weighted imaging, proton therapy

## Abstract

**Background:**

Glioblastoma is the most aggressive brain tumor, with a 5-year survival rate of only 8%. Radiotherapy plays a crucial role in multimodal treatment. With novel therapy approaches emerging, we evaluated specific imaging patterns of progression in patients treated with a sequential proton boost after standard radiochemotherapy.

**Methods:**

We analyzed data from 144 patients with histologically confirmed glioblastoma (CNS-WHO grade4), 94 received a sequential proton boost, and 50 underwent conventional photon radiation. Tumor progression (TP) was confirmed histologically or according RANO criteria. MRI segmentation was automated using CE-marked AI software, tumor voxels were classified by location (inside or outside the clinical target volume [CTV]) and CBV-perfusion status (inclusion-zone).

**Results:**

At progression, the proton group showed significantly larger contrast-enhancing tumor volumes outside the CTV (*P* = .007), focused in the CTV-PTV-transition-zone (*P* = .004) with no significant difference inside the CTV (*P* = .43). Subgroup analysis revealed greater extra-CTV volumes in MGMT-unmethylated (*P* = .019), TERT-mutant (*P* = .004), and RTK II subtype patients (*P* = .044). Metabolically active tumor tissue showed no significant differences between groups, inside or outside the CTV (*P* = .15 and .30).

**Conclusion:**

Sequential proton boost therapy was associated with a distinct spatial pattern of radiographic progression, characterized by increased contrast enhancement beyond the CTV, especially in the CTV-PTV-transition-zone, without a corresponding rise in metabolic activity. These findings are hypothesis-generating and may reflect radiobiological range-edge effects or treatment-related tissue responses rather than true TP. Prospective studies integrating dosimetric and metabolic data are warranted to elucidate underlying mechanisms.

Key Points

- Sequential proton boost vs conventional photon radiotherapy: greater out-of-CTV enhancement at progression
- Metabolically active tumor burden comparable between modalities
- Effects strongest in genetically aggressive tumor subtypes

Importance of the Study

Glioblastoma remains a highly aggressive brain tumor with limited survival despite therapeutic advances. Proton radiotherapy provides superior dose distribution compared with conventional photons. Distinct post-treatment imaging patterns have been described after proton therapy, but their clinical relevance remains uncertain. We compared sequential proton boost with conventional photon-based radiotherapy in 144 patients using fully automated, clinically integrated AI-based MRI segmentation and perfusion-weighted imaging.

At radiographic progression, proton-treated patients showed greater contrast-enhancing tumor volume outside the clinical target volume, including in MGMT-unmethylated and TERT-mutant tumors, while metabolically active tumor burden remained comparable between modalities. This dissociation between morphologic enhancement and metabolic activity may indicate that part of the peripheral enhancement reflects treatment-related tissue response rather than active tumor infiltration. These observations are hypothesis-generating and suggest that future studies should integrate dosimetric and metabolic data to clarify underlying mechanisms and inform target delineation in proton therapy.

Glioblastomas are a subgroup of primary brain tumors with a highly infiltrative and genetically heterogeneous nature. Despite advances in treatment, GBM remains a devastating diagnosis, with a 5-year survival rate of only 8%.^1^ The standard of care includes surgical resection followed by radiochemotherapy (RCT). This typically involves photon-based radiotherapy with concurrent Temozolomide (TMZ), the Stupp-Scheme, or with TMZ and Lomustine (CCNU), referred to as the CeTeG-Scheme.^2^^,^^3^ Recent advances in GBM treatment include immunotherapy, tumor-treating fields (TTF), and oncolytic viruses (OVs), as well as innovations in radiotherapy using techniques like particle therapy and intensity-modulated radiation therapy (IMRT). Charged particle therapy enhances localized tumor control through unique physical and biological properties. Its favorable dose distribution allows high doses to be delivered to tumors while sparing surrounding, normal tissue, due to a sharp energy peak known as the Bragg peak.^4–6^

Contrast-enhanced magnetic resonance imaging (MRI) is the primary imaging modality assessing the extent of tumor resection, monitoring treatment response, and detecting disease progression.^7^ Differentiating pseudoprogression (PsP) from true tumor progression (TP) is critical during follow-up. PsP represents therapy-related changes (TRC) that mimic TP on imaging, often seen as an increase in contrast-enhancing lesions and perifocal edema, which later stabilize or resolve.^8–10^ Current meta-analyses show that approximately 60% of patients develop PsP after RT.^11^ Differentiating these TRCs from true TP is decisive in further treatment of the patient as these Imaging findings guide alternative therapy approaches, repeated resection or continuation of current therapy in hope of resolution.

As therapy with charged particles is still a new and scarcely available therapeutic option, several authors described distinct imaging features attributed to this novel therapy approach.^12^ Our study aims to further characterize these changes by visualizing distinct spatial patterns of progression in patients treated with sequential proton-boosted RT compared to patients treated with conventional photon-based RT.

## Methods

### Patients and Radiotherapy

We performed a retrospective analysis of 144 patients with CNS-WHO Grade 4 Glioblastoma who received treatment from 01/2018–12/2022 and follow-up imaging between 01/2018–12/2024 at the University Hospital Giessen. Patients had primary resection followed by concomitant RCT using established STUPP- or CeTeG-protocols.

### Photon Radiotherapy

A total of 50 patients received volumetric arc therapy with daily image guidance via cone-beam CT (VMAT/IGRT) planned on co-registered T2-FLAIR and contrast-enhanced T1-weighted MRI. A total dose of 60 Gy in 30 fractions (2 Gy/fraction) was delivered according to contemporary ESTRO guidelines.^13^ 60 Gy were prescribed to the resection cavity, with optional inclusion of peritumoral edema and a 2 cm clinical target volume (CTV) margin was applied.

### Proton Radiotherapy

A total of 94 patients received a sequential proton boost following 50 Gy photons delivered according to contemporary ESTRO guidelines, as described above. The proton component was planned analogous to the CLEOPATRA trial, with 5 mm CTV and 3 mm PTV margins.^14^^,^^15^ A dose of 10 Gy (RBE) was applied using pencil beam (active raster) scanning via 2 horizontal beams, encompassed by the 95% isodose, in 2 Gy (RBE)/fraction under daily image guidance.

### Image Acquisition

During regular follow-up, patients had multiparametric MRI examinations at 1.5T or 3.0T (Magnetom Espree/Skyra, Siemens Healthineers, Erlangen, Germany) with a dedicated tumor protocol including perfusion-weighted imaging (PWI). Progression was determined histologically or according to the modified RANO criteria.^16^

### Post-Processing

#### Tumor Segmentation and Analysis

Longitudinal MRI scans were analyzed using an AI-based segmentation software (mdbrain, mediaire GmbH, Berlin, Germany). This CE-labeled solution employs a U-Net convolutional neural network (CNN) to automatically segment tumors into 3 distinct compartments: contrast-enhancing tumor, non-contrast-enhancing tumor, and peritumoral edema. The software generates 3 outputs: (1) a DICOM segmentation file with color-coded visualization of tumor compartments, (2) a volumetric report quantifying each compartment, and (3) a segmentation mask containing static voxel values for each tumor component.

#### Clinical Target Volume and Progression Analysis

In the postoperative MRI, we constructed a convex hull around the resection cavity to define the CTV with concentric expansion by 5 mm to resemble the planning target volume (PTV), according to ESTRO guidelines. Exemplary images are provided in the Supplementary Material. The ESTRO guidelines recommend reduced margins to anatomical barriers such as the skull or the Falx. However, for computational purposes, the convex hull generated extends beyond these anatomical boundaries.

TP was determined according to the modified Response Assessment in Neuro-Oncology (mRANO) criteria.^16^ All cases were independently reviewed by 2 blinded neuroradiologists, with discrepancies resolved by the multidisciplinary neuro-oncology tumor board, which served as the reference standard. When available, histopathological confirmation from re-resection was used to differentiate true progression from treatment-related changes.

At the time of TP, we analyzed the spatial distribution of recurrent tumor relative to the CTV. Additionally, we defined an inclusion zone (IZ) based on PWI, which represented metabolically active tumor tissue. Quantitative assessment included measurement of contrast-enhancing tumor voxels in 6 regions: (1) Inside the CTV, (2) Outside the CTV, (3) within the IZ inside the CTV, (4) within the IZ outside the CTV, (5) in the CTV-PTV transition Zone, and (6) outside the PTV.

Statistical analysis was performed using Python and R, with statistical significance set at *P ≤ .*05.^17^^,^^18^ Normal distribution was tested with Shapiro–Wilks test. T-test and Mann–Whitney U *t*est were used when applicable. Categorical variables were compared using Chi-squared test (χ^2^).

## Results

### Patients

We analyzed 144 patients with Glioblastoma IDH-wildtype (CNS-WHO grade 4), 94 received sequential proton boost and 50 conventional photon radiation. Median age at presentation was 62 (IQR: 13) in the proton-boost-group and 63 (IQR: 15.5) in the photon-group.

In the proton-group MGMT-Promoter methylation was present in 54 patients, absent in 24 and unknown in 16. TERT-Promoter methylation was present in 64 patients, absent in 13, and unknown in 17. TTF were used in 17 Patients, not used in 41 and 36 with unknown status.

In the photon-group MGMT-Promoter methylation was present in 30 patients and absent in 8 with 12 having unknown status. TERT-Promoter methylation was present in 31 Patients and absent in 6 with 13 having unknown status. TTF were used by 6 patients, not used in 23 and 21 with unknown status.

Methylation subtyping showed RTK II as the most common subtype (30 proton, 10 photon), followed by MES (20 proton, 9 photon) and RTK I (15 proton, 7 photon). 14 patients (7 in each group) had no determined subtype. Detailed demographics are summarized in Table 1.

**Table 1.** Detailed patient characteristics, molecular features, and therapy details for the patient groups treated with sequential proton boost and conventional photon radiotherapy

Variable	Proton group (*n* = 94)	Photon group (*n* = 50)	*P* value
Median age in years (IQR)	62 (IQR: 13)	63 (IQR: 15.5)	.364
MGMT-promoter status, *n* (%)			
Present	54 (56.8%)	30 (58.8%)	.111
Absent	24 (25.3%)	8 (15.7%)	
Unknown	16 (17.0%)	12 (24.0%)	.449
TERT-promoter status, *n* (%)			
Present	64 (67.4%)	31 (60.8%)	.623
Absent	13 (13.7%)	6 (11.8%)	
Unknown	17 (17.9%)	13 (26.0%)	.514
TTF usage, *n* (%)			
Used	17 (17.9%)	6 (11.8%)	.474
Not used	41 (43.2%)	23 (45.1%)	
Unknown	36 (38.3%)	21 (42.0%)	.846
Molecular subtype, *n* (%)			
RTK II	30 (31.6%)	10 (19.6%)	.136
MES	20 (21.1%)	9 (17.6%)	1
RTK I	15 (15.8%)	7 (13.7%)	.824
No subtype	7 (7.4%)	7 (13.7%)	.419
Unknown	22 (23.2%)	17 (33.3%)	.300
Chemotherapy, *n* (%)			
CeTeG	24 (25.5%)	10 (20%)	.665
TMZ	61 (64.9%)	30 (60%)	.663
Unknown	9 (10.0%)	10 (20%)	.083
Number of chemotherapy cycles; median (IQR)	5 (2.2-6.0)	3 (2-6)	.324

### Progression Free Survival

TP occurred in 79 out of 94 proton-patients (84.0%) and 40 out of 50 photon-patients (80.0%). Median Progression-free survival was 9.56 months in the proton group vs 6.77 months in the photon group (*P *=* .1160*).

### Imaging Analysis

#### Proton vs Photon-Radiotherapy

**Figure 1.**
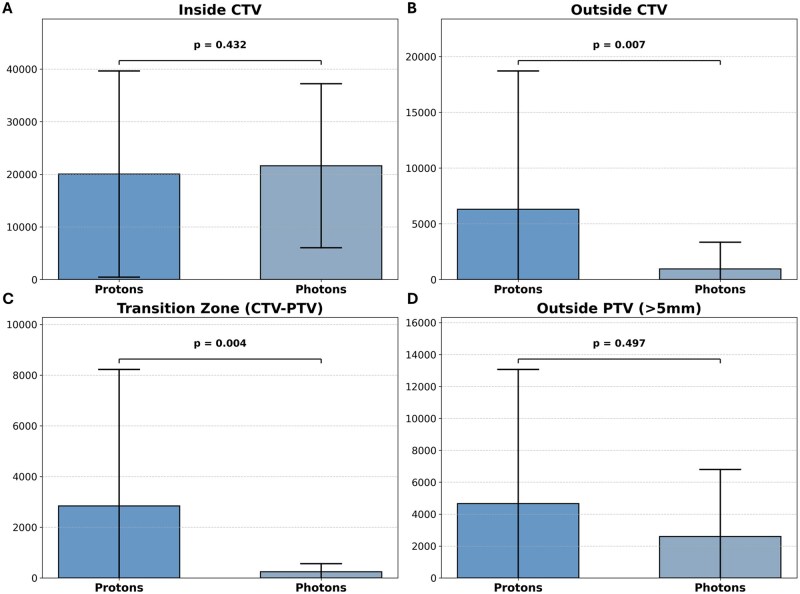
Bar charts comparing the number of contrast enhancing voxels at radiographic progression between sequential proton-boost and conventional photon radiotherapy by spatial location relative to the treatment volume; inside CTV (A), outside CTV (B), CTV-PTV margin zone (C), and outside PTV (D).

Absolute voxel count was significantly higher outside the CTV in the proton group (median = 2257, IQR = 249-6682) compared to the photon group (median = 156, IQR = 34-368; *P* = .007). No significant difference was found inside the CTV (protons: median = 14489, IQR = 6124-28218; photons: median = 19120.5 IQR = 8141.8-33574.8; *P* = .432). In the CTV-PTV transition zone we observed a significantly higher recurrence voxel count in the proton group (median = 1475, IQR = 192-2345) compared to the photon group (median = 156, IQR = 33.5-261; *P* = .004). No significant difference was found beyond the PTV margin (protons: median = 2392, IQR = 477-4794; photons: median = 274, IQR = 183-3905; *P* = .497) (Table 2 and Figure 1).

**Table 2.** Comparison of voxel counts between Proton and Photon groups showing statistical difference of tumor voxels outside the clinical target volume and in the CTV-PTV transition zone

Region	Proton group	Photon group	*P* value
Inside CTV	Median: 14 489.0 IQR: 6124-28 218	Median: 19120.5 IQR: 8141.8-33574.8	.432
Outside CTV	Median: 2257 IQR: 249-6682	Median: 156 IQR: 34-368	.007*
CTV-PTV transition zone	Median: 1475.0 IQR: 192.0-2345.0	Median: 156.0 IQR: 33.5-261.0	.004*
Outside PTV	Median: 2392 IQR: 477-4794	Median: 274.0 IQR: 183.0-3904.5	.497
Inside IZ and inside CTV	Median: 1382 IQR: 639-4440	Median: 3221.5 IQR: 1787.5-7949.2	.145
Inside IZ and outside CTV	Median: 802 IQR: 491-2308	Median: 13 IQR: 9-3707	.303

*Statistically significant (*p* < 0.05).

**Figure 2.**
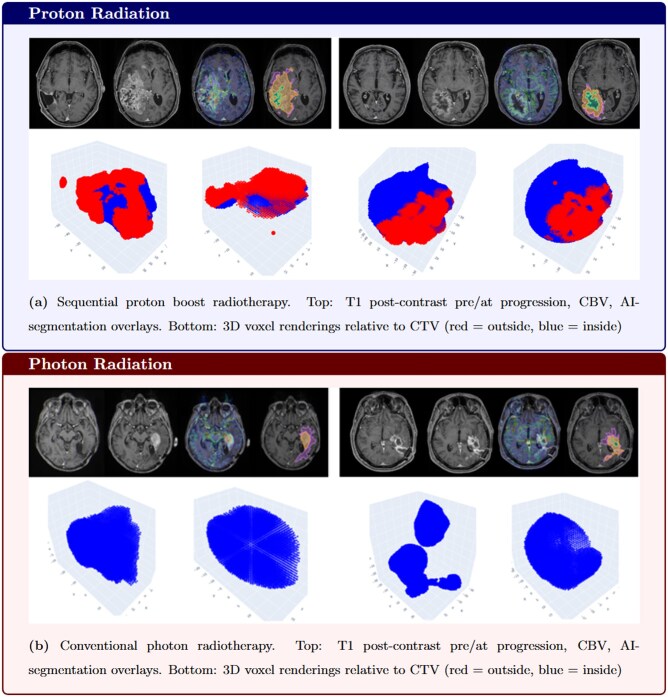
Proton radiotherapy is associated with significantly more contrast enhancing voxels outside the clinical target volume and in the clinical target volume to planning target volume margin zone compared with photon radiotherapy. No significant differences in the clinical target volume or outside the planning target volume.

For metabolically active tumor in the IZ, no significant differences were observed either inside (protons: median = 1382, IQR = 639-4440; photons: median = 3221.5, IQR = 1787.5-7949.2; *P* = .15) or outside the CTV (protons: median = 802, IQR = 491-2308; photons: median = 13, IQR = 9-3707; *P* = .30). An example is given in Figure 2.

To further analyze the spatial distribution of recurrence relative to the CTV, voxels were categorized by distance from the CTV margin.

In the photon group, 42.48% of recurrence voxels were located within ≤2 cm of the CTV, 39.28% between 2 and 3 cm, and 18.24% beyond 3 cm. In the proton group, 36.58% of voxels were within ≤2 cm, 33.55% between 2 and 3 cm, and 29.86% beyond 3 cm from the CTV. No statistically significant differences were observed between treatment modalities across the distance categories (*P* = .86 for ≤2 cm, *P* = .41 for 2–3 cm, and *P* = .93 for >3 cm). A detailed summary is provided in the Supplementary Material.

#### Subanalysis by Mutation Status

Patients with positive methylation status of the MGMT-promoter showed no significant differences in voxel counts inside the CTV, with patients receiving proton-RT showing a median of 14491.5 voxels (IQR: 6484.25-30 496.5) and photon-treated patients a median of 16 391.5 (IQR: 6649.5-33 274.25; *P* = .755) at time of progression. Similarly, in MGMT unmethylated patients, median voxel counts within the CTV were comparable between protons (14 509; IQR: 6826-23 807.5) and photons (23 861.5; IQR: 14 135.25-34 051.25; *P* = .470).

Voxel counts outside the CTV were also not significantly different for MGMT methylated patients (protons: 2045; IQR: 173-7082 vs photons: 156; IQR: 57-359; *P* = .095). However, a significant difference was detected in MGMT unmethylated patients outside the CTV, with higher voxel counts in the proton group (median: 3843; IQR: 1837-3985) compared to the photon group (median: 144; IQR: 17-470.5; *P* = .019).

No significant differences were observed within the CTV across TERT mutations (protons: 14 514; IQR: 7809–28 927 vs photons: 26 967; IQR: 15 500.75-35 847.5; *P* = .099) or TERT nonmutant cases (protons: 9593; IQR: 6082.75-29 483.25 vs photons: 2068; IQR: 1183.75-7474.5; *P* = .154).

Outside the CTV, TERT mutant patients showed a significantly higher voxel count median: 2098.5; IQR: 248.75-6681.5) compared to photons (median: 110.5; IQR: 41.25-305.5; *P* = .004). No significant difference was found in TERT non-mutants (protons: 1839.5; IQR: 418.5-3472.75 vs photons: 270; IQR: 137.5-4200.5; *P* = .960).

Within the CTV, no significant differences were found between protons and photons across molecular subtypes: RTK II (protons: 13 106.5; IQR: 6032-28 690.5 vs photons: 15 725; IQR: 8478-24725; *P* = .969), MES (protons: 16,459; IQR: 9831–27 589 vs photons: 23 861.5; IQR: 19 743.75-29 986.5; *P* = .393), or RTK I (protons: 22 852.5; IQR: 10 609.75-36 424.25 vs photons: 11 437.5; IQR: 5089-21 932.25; *P* = .535).

**Figure 3.**
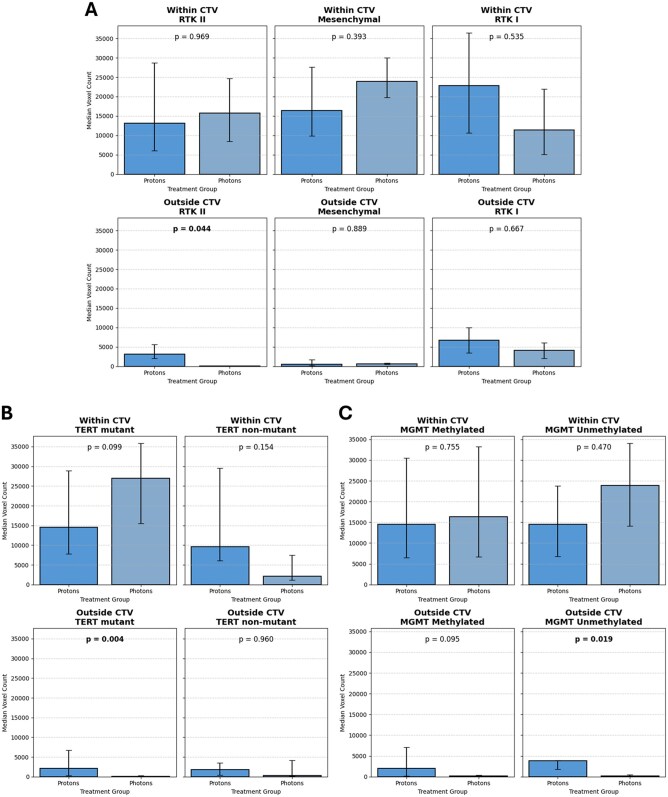
Bar charts comparing sequential proton-boost radiotherapy and conventional photon radiotherapy at radiographic progression, stratified by (A) methylation subclasses, (B) MGMT promoter methylation status, and (C) TERT promoter methylation status. Counts are shown by location relative to the clinical target volume (within vs outside).

Outside the CTV, RTK II subtypes exhibited significantly higher voxel counts in the proton group (median: 3129; IQR: 1993-5677.25) compared to photons (median: 80.5; IQR: 42.75–118.25; *P* = .044), while MES (protons: 547; IQR: 216.5-1695 vs photons: 671; IQR: 470.5-871.5; *P* = .889) and RTK I (protons: 6743.5; IQR: 3479.25-10 007.75 vs photons: 4074.5; IQR: 2046.25-6102.75; *P* = .667). Other subtypes did not show significant differences (Table 3 and Figure 3).

**Table 3.** Subgroup analysis of absolute voxel count within and outside the CTV across molecular and clinical biomarkers

Subgroup	Region	Proton median (IQR)	Photon median (IQR)	*P* value
MGMT methylated	Within CTV	14 491.5 [6484.3-30 496.5]	16 391.5 [6649.5-33 274.3]	.755
MGMT unmethylated	Within CTV	14 509 [6826-23 807.5]	23 861.5 [14 135.3-34 051.3]	.470
MGMT methylated	Outside CTV	2045 [173-7082]	156 [57-359]	.095
MGMT unmethylated	Outside CTV	3843 [1837-3985]	144 [17-470.5]	.019*
TERT mutant	Within CTV	14 514 [7809-28 927]	26 967 [15 500.8-35 847.5]	.099
TERT nonmutant	Within CTV	9593 [6082.8-29 483.3]	2068 [1183.8-7474.5]	.154
TERT mutant	Outside CTV	2098.5 [248.8-6681.5]	110.5 [41.3-305.5]	.004*
TERT nonmutant	Outside CTV	1839.5 [418.5-3472.8]	270 [137.5-4200.5]	.960
RTK II	Within CTV	13 106.5 [6032-28 690.5]	15 725 [8478-24725]	.969
Mesenchymal	Within CTV	16 459 [9831-27 589]	23 861.5 [19 743.8-29 986.5]	.393
RTK I	Within CTV	22 852.5 [10 609.8-36 424.3]	11 437.5 [5089-21 932.3]	.535
RTK II	Outside CTV	3129 [1993-5677.3]	80.5 [42.8-118.3]	.044*
Mesenchymal	Outside CTV	547 [216.5-1695]	671 [470.5-871.5]	.889
RTK I	Outside CTV	6743.5 [3479.3-10 007.8]	4074.5 [2046.3-6102.8]	.667

*Statistically significant (*p* < 0.05).

### Limitations

This study has several limitations that should be acknowledged. Most importantly, detailed dosimetric data, including original treatment plans and dose-volume histograms, were not available for this retrospective cohort. The CTV and PTV were therefore manually reconstructed based on contemporary delineation guidelines and may not precisely reflect the actual planning volumes used for each individual patient. As a result, we were unable to quantitatively evaluate the relationship between dose distribution and the observed enhancement patterns. Without voxel-wise dose information, any mechanistic interpretation of peripheral enhancement must therefore be considered hypothesis-generating.

Second, the uneven sample sizes between the proton and photon therapy groups may introduce bias and reduce the robustness of intergroup comparisons. Additionally, the single-center, retrospective design limits the generalizability of the findings. The availability of molecular data, such as MGMT- and TERT-promoter mutations, was incomplete for some patients, which may have influenced subgroup analyses and limited insight into these molecular factors.

A further limitation relates to the use of an AI-based tumor segmentation tool. Although all segmentations were manually reviewed and corrected when necessary, minor inaccuracies or segmentation biases cannot be completely excluded and may have influenced volumetric measurements.

Finally, the absence of randomization and the requirement for patients receiving proton therapy to travel to the proton facility may have introduced selection bias, potentially favoring individuals with higher performance status.

## Discussion

Identification of TP in glioblastoma follow-up remains of central importance for guiding subsequent therapeutic strategies. However, distinguishing true TP from therapy-related effects, such as PsP or radionecrosis, remains a significant diagnostic challenge. This difficulty is particularly relevant with newer therapy modalities such as small molecules and charged particle therapy, which are associated with distinct imaging characteristics not commonly observed following conventional RCT. Proton therapy has already been associated with distinct radiographic patterns. Prior studies have reported a delayed onset of PsP and contrast enhancement occurring at anatomical sites more distant from the original tumor location compared to photon-based therapy.^12^ To enable a more accurate and reliable differentiation between therapy-related changes and true progression, specialized imaging techniques, including FET-PET-CT, PWI, or ASL-perfusion, are commonly employed. A recent meta-analysis showed comparable diagnostic performance across these imaging modalities, with sensitivities of 90% for DCE/DSC perfusion, 89% for FET-PET, and 86% for ASL-perfusion in distinguishing true progression from therapy-related changes.^19^ Accurate differentiation is crucial, as it directly influences therapeutic decisions whether to continue the current regimen, to proceed with surgery or to stop therapy.

Our analysis revealed a distinct spatial pattern at the time of radiologically confirmed progression in patients treated with sequential proton boost therapy. These patients exhibited significantly larger tumor volumes outside the CTV compared to conventional photon-treated patients (*P *=* .*007), while no significant difference was observed within the CTV (*P *=* .*432). Further subdividing this analysis and incorporating the PTV showed that this effect is most concentrated in the CTV-PTV transition zone where voxel counts were significantly higher in the group receiving sequential proton boost (*P = .*004). Beyond the PTV the number of voxels in the 2 patient groups was not significantly different. Importantly, this analysis was conducted at the time of radiologically confirmed progression, which in the majority of cases occurred months after completion of radiation therapy, rather than during or shortly after treatment.

The observed differences in tumor volumes between treatment modalities may reflect a complex interaction between dose distribution and tissue response rather than a single underlying mechanism. Two complementary hypotheses may explain this finding.

First, the pattern may primarily represent a treatment-related tissue response. The steep dose gradient at the distal end of the proton range (Bragg peak) can induce localized vascular and inflammatory changes that manifest as delayed contrast enhancement without corresponding increases in perfusion or metabolic activity. The absence of significant differences in perfusion-PWI (*P* = .15; *P* = .303) supports this interpretation, suggesting that the peripheral enhancement is more consistent with a radiation-induced effect than viable tumor growth.

Second, it remains possible that microscopic infiltrative tumor cells extending beyond the CTV received subtherapeutic doses due to the sharp proton dose falloff, whereas photon therapy’s more gradual dose gradient might incidentally treat these peripheral regions with intermediate doses.^4^^,^^20^ Although range-edge underdosage remains a plausible contributing factor, the lack of a corresponding increase in perfusion metrics indicates that non-viable tissue response rather than active tumor proliferation may predominate in these regions.

Subgroup analysis provided further insight into this phenomenon and supports our hypothesis. In MGMT-unmethylated and TERT-mutant patients, both typically associated with poorer prognosis and more aggressive tumor biology, significantly greater tumor volumes were observed outside the CTV in the proton group compared to the photon group (*P *=* .*019 and 004, respectively). Similarly, the RTK II molecular subtype also demonstrated significantly larger volumes outside the CTV with proton therapy (*P *=* .*044). Our findings suggest that the observed spatial patterns may be more pronounced in molecularly aggressive tumor subtypes, which typically harbor more extensive microscopic infiltration and may exhibit stronger inflammatory response to residual disease. Importantly, no significant differences were observed within the CTV across molecular subgroups, supporting the hypothesis that local tumor control within the primary target volume remains effective.

Despite these spatial differences in progression patterns, the proton group exhibited a longer median progression-free survival (PFS) compared to the photon group (9.56 months vs 6.77 months, respectively), although the difference did not reach statistical significance (*P *=* .*116).

Subgroup analysis reinforced the prognostic relevance of established molecular alterations across both treatment modalities. MGMT-promoter methylation was associated with longer PFS in both the proton and photon therapy groups, with trends approaching statistical significance (Proton: 9.97 vs 7.46 months, *P* = .0757; Photon: 7.44 vs 4.30 months, *P* = .0942).^21–25^

Similarly, our findings for TERT-promoter methylation were consistent with prior reports, demonstrating an association with poorer prognosis.^26^^,^^27^ In the proton therapy cohort, patients harboring the C228T mutation experienced significantly shorter PFS compared to those without the mutation (7.74 vs 14.03 months, *P* = .0478). In contrast, the photon group showed a less marked difference, potentially due to limited sample size.

The observed spatial patterns of progression, particularly the tendency for contrast enhancement to occur outside the CTV, particularly in the CTV-PTV transition zone, in proton-treated patients, may represent a distinct imaging phenotype associated with particle therapy. The location of recurrence relative to the CTV could serve as an exploratory imaging biomarker in future studies, providing insights into treatment response and radiobiological range-edge effects. While these findings raise the possibility that CTV margin definitions and proton dose fall-off characteristics influence spatial progression patterns, the absence of detailed dosimetric data precludes definitive conclusions. Future research should therefore incorporate comprehensive dose-distribution analyzes, advanced metabolic imaging, and histopathologic validation to elucidate the biological mechanisms underlying these observations and assess their implications for proton therapy planning.

In summary, this study provides a descriptive characterization of distinct spatial progression patterns in glioblastoma following sequential proton boost therapy. The increased contrast enhancement observed outside the CTV, without corresponding increases in metabolic activity, highlights the need for cautious interpretation of post-treatment imaging in proton-treated patients. These findings are hypothesis-­generating, underscoring the need for prospective studies integrating dosimetric data, advanced imaging, and tissue correlation. Such analyses will be crucial to determine whether the observed enhancement patterns reflect microscopic tumor infiltration, radiobiological range-edge effects, or treatment-related tissue responses, and to guide future target definition and dose-optimization strategies in proton therapy.

## Supplementary Material

vdag116_Supplementary_Data

## Data Availability

Data or evaluation code will be made available upon reasonable request.
