# Supplementary material for: Imaging patterns of glioblastoma progression: Comparative analysis of sequential proton boost versus conventional photon therapy
Source: Neurooncol Adv. 2026 May 4;8(1):vdag116. doi: 10.1093/noajnl/vdag116 (PMC13199058; doi:10.1093/noajnl/vdag116)
Supplement: vdag116_Supplementary_Data [file vdag116_supplementary_data.zip › Supplementary Table and Figure Legends.docx]

| **Sequence** | **Scanning Sequence** | **Mode** | **Slice Thickness (mm)** | **Repetition Time (ms)** | **Echo Time (ms)** | **Inversion Time (ms)** | **Acquisition Matrix** |
| --- | --- | --- | --- | --- | --- | --- | --- |
| T1-pre | SE | 2D | 4 | 615 | 8.80 | None | [0, 256, 256, 0] |
| FLAIR | [SE, IR] | 2D | 4 | 9000 | 92.00 | 2500 | [0, 256, 224, 0] |
| T2* | GR | 2D | 4 | 1190 | 25.60 | None | [0, 256, 256, 0] |
| DSC-Perfusion | EP | 2D | 5 | 1440 | 51.00 | None | [128, 0, 0, 128] |
| EPI-DWI | EP | 2D | 4 | 6900 | 113.00 | None | [192, 0, 0, 192] |
| T2 | SE | 2D | 4 | 8120 | 83.00 | None | [0, 384, 324, 0] |
| T1 post contrast | [GR, IR] | 3D | 1 | 1960 | 2.86 | 900 | [0, 256, 246, 0] |

**Supplementary Table 1:** MRI Sequence Parameters for 1.5 Tesla

| **Sequence** | **Scanning Sequence** | **Mode** | **Slice Thickness (mm)** | **Repetition Time (ms)** | **Echo Time (ms)** | **Inversion Time (ms)** | **Acquisition Matrix** |
| --- | --- | --- | --- | --- | --- | --- | --- |
| T1-pre | GR | 2D | 4 | 227 | 10.1 | None | [0, 320, 256, 0] |
| FLAIR | [SE, IR] | 2D | 4 | 9000 | 90 | 2500 | [0, 320, 224, 0] |
| SWI | GR | 2D | 16 | 27 | 20 | None | [0, 256, 223, 0] |
| DSC-Perfusion | EP | 2D | 4 | 1600 | 30 | None | [128, 0, 0, 128] |
| EPI-DWI | EP | 2D | 4 | 5500 | 65 | None | [160, 0, 0, 160] |
| T2 | SE | 2D | 4 | 6900 | 100 | None | [0, 512, 384, 0] |
| T1-post contrast | [GR, IR] | 3D | 1 | 2100 | 2.58 | 900 | [0, 256, 256, 0] |

**Supplementary Table 2:** MRI Sequence Parameters for 3.0 Tesla

| **Group** | **Distance Category** | **Voxel Count** | **Proportion (%)** | **p-value** |
| --- | --- | --- | --- | --- |
| Photons | ≤2 cm | 164,851 | 42.48 | 0.86 |
| Protons | ≤2 cm | 445,870 | 36.58 |  |
| Photons | 2–3 cm | 145,823 | 39.28 | 0.41 |
| Protons | 2–3 cm | 408,960 | 33.55 |  |
| Photons | >3 cm | 47,770 | 18.24 | 0.93 |
| Protons | >3 cm | 363,964 | 29.86 |  |

**Supplementary Table 3**: Distribution of recurrence voxels by distance from the resection cavity for patients treated with photon radiotherapy or sequential proton boost.


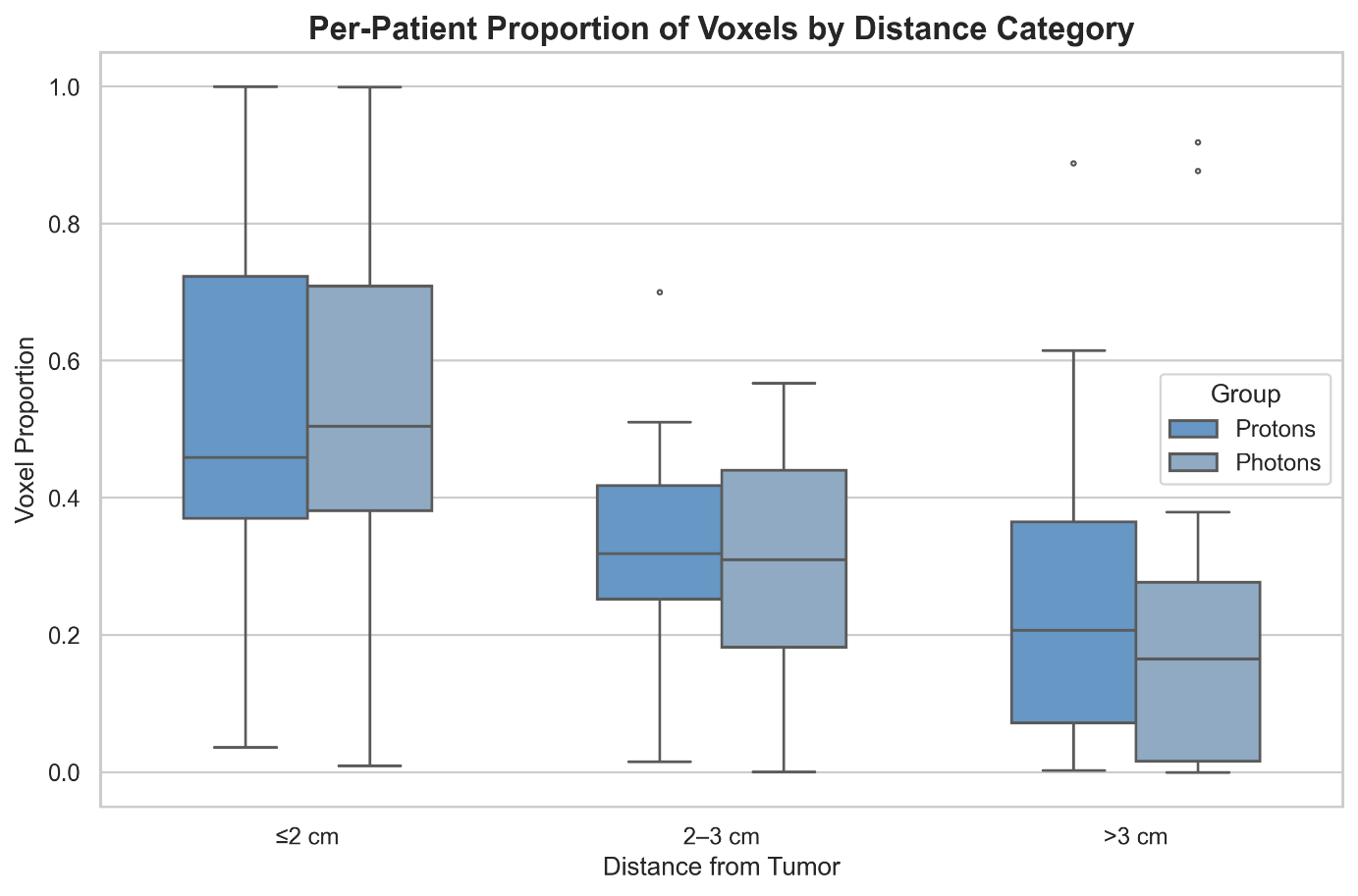
**Supplementary Figure 1**: Boxplots showing the distribution of contrast enhancing voxels by distance from the resection cavity for patients treated with photon radiotherapy or sequential proton boost.


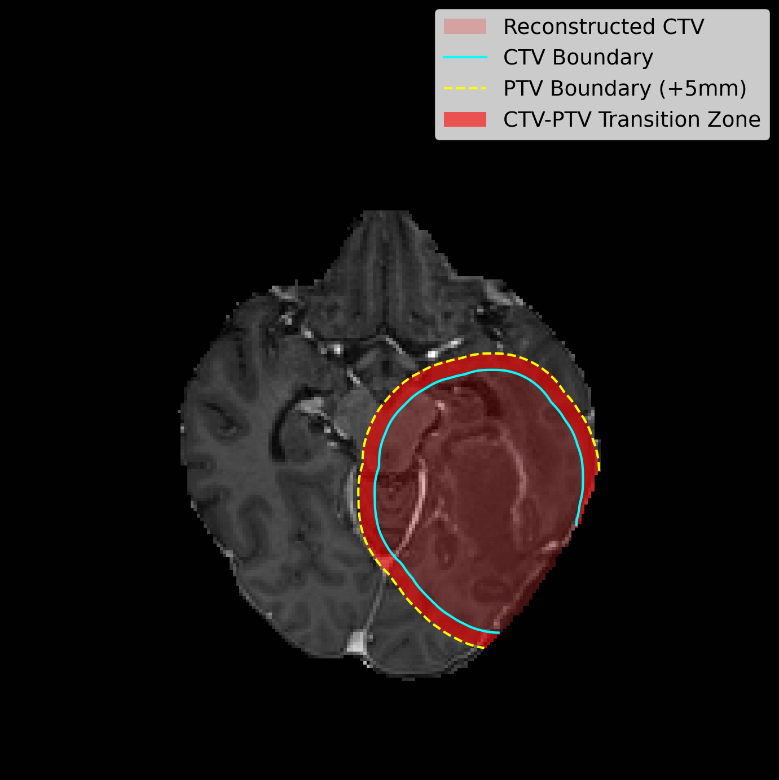

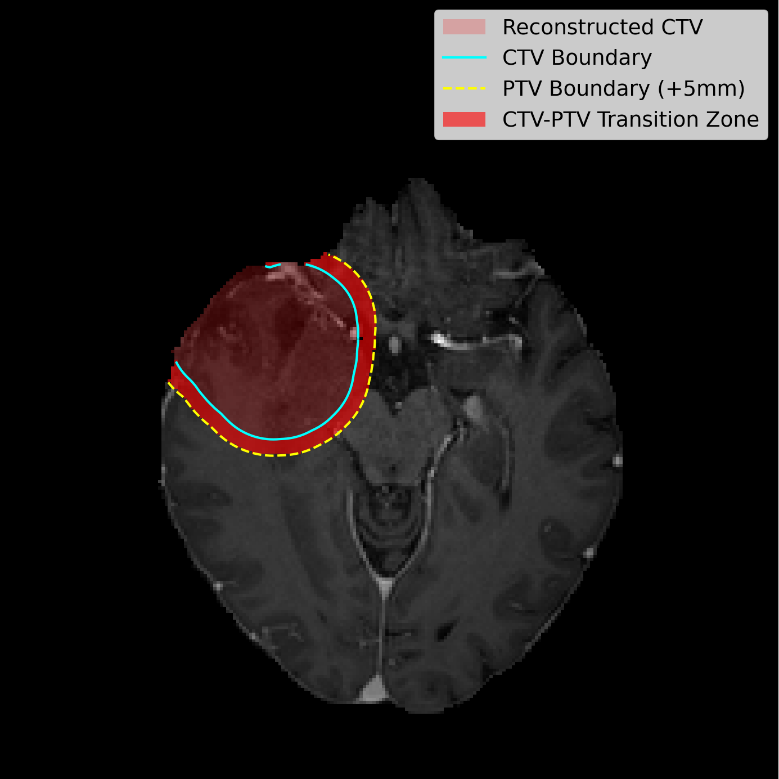


**Supplementary Figure 2:** Exemplary images of reconstructed CTV and PTV in two patients.
